# Pediatric Theranostics in a 13-Year-Old Female with Bronchial Carcinoid

**DOI:** 10.1055/s-0045-1809341

**Published:** 2025-05-26

**Authors:** Justine Trpezanovski, Jonathan Karpelowsky, Elizabeth Hesketh, Kevin London

**Affiliations:** 1 Department of Nuclear Medicine, The Children's Hospital at Westmead, Sydney Children's Hospitals Network, Westmead, Sydney, NSW, Australia; 2 Department of Paediatric Surgery, The Children's Hospital at Westmead, Sydney Children's Hospitals Network, Westmead, Sydney, NSW, Australia; 3 Discipline of Child and Adolescent Health, Sydney Medical School, Faculty of Medicine and Health, University of Sydney, Sydney, NSW, Australia; 4 Department of Paediatric Oncology & Haematology, John Hunter Children's Hospital, Newcastle, NSW, Australia; 5 Discipline of Medical Imaging Science, Sydney School of Health Sciences, Faculty of Medicine and Health, University of Sydney, Sydney, NSW, Australia; 6 Alfred Nuclear Medicine and Ultrasound, Royal Prince Alfred Hospital Medical Centre, Newtown, Sydney, NSW, Australia

**Keywords:** bronchial, carcinoid, LuTATE, neuroendocrine, pediatrics

## Abstract

Pediatric bronchial carcinoid tumors are rare, accounting for a significant proportion of primary lung tumors in children but only a small fraction in adults. These tumors can present with symptoms such as cushing's syndrome due to ACTH secretion. Complete surgical resection typically results in favorable outcomes, with most tumors expressing somatostatin receptors, making them amenable to peptide receptor radionuclide therapy (PRRT) with (177Lu)Lu-DOTA-TATE (LuTATE).

This case report describes a 13-year-old female with a bronchial carcinoid tumor treated with multi-cycle high-dose LuTATE therapy in the neoadjuvant setting. Initial imaging and biopsy confirmed a grade G1 pulmonary carcinoid with intense somatostatin receptor expression. The patient underwent two cycles of LuTATE, with dosimetry calculations guiding dose escalation while maintaining safe kidney radiation exposure. Posttherapy scans showed a significant metabolic response of suspected nodal metastases and evidence of partial response of the primary tumor.

Two further LuTATE cycles were administered, with continued monitoring of kidney dosimetry to ensure safety. The treatment was well-tolerated, and the patient showed no significant complications. The case highlights the potential of LuTATE therapy to downstage tumors and reduce surgical morbidity in pediatric patients. Given the rarity of pediatric bronchial carcinoid tumors, phase III clinical trials are unlikely, but this report supports the inclusion of LuTATE in multidisciplinary treatment planning.

In conclusion, LuTATE therapy, guided by dosimetry calculations, offers a valid treatment option for pediatric bronchial carcinoid tumors, balancing efficacy, and safety in a challenging clinical scenario.

## Introduction

Theranostics has been part of nuclear medicine practice since the 1950s when iodine-131 was reported for the treatment of thyroid cancer. The 1980s saw the introduction of I-131 MIBG to treat pediatric neuroblastoma. Subsequent development of new peptides and increased accessibility to different radionuclides provide the potential to implement the theranostic technique earlier into treatment regimens.

Peptide receptor radionuclide therapy (PRRT) using the theranostic pairing of (68Ga)Ga-DOTA-TATE (GaTATE) and (177Lu)Lu-DOTA-TATE (LuTATE) has an established role in treating adults with neuroendocrine tumors but its role in pediatrics is not well studied. There are few published studies describing the utility of LuTATE therapy in children with neuroblastoma and its use in other pediatric neuroendocrine tumors is unclear.

We report a rare case of pediatric bronchial carcinoid tumor treated with LuTATE in the neoadjuvant setting. Kidney radiation dosimetry was used to support multiple cycles of LuTATE therapy while ensuring kidney radiation exposure was maintained within safe dose constraints. The LuTATE therapy appeared to provide a treatment response allowing a down-staged surgical approach potentially reducing surgical morbidity.

## LuTATE Administration

Our LuTATE therapy protocol involves administering two cycles of LuTATE each of which has dosimetry calculations performed to estimate kidney radiation dose. Baseline blood tests (full blood count, liver function tests, electrolytes, urea, and creatinine) and a radionuclide GFR are performed and used to assess for the need to reduce LuTATE dosing if there is preexisting renal or bone marrow impairment. The first LuTATE cycle is an empiric dose of 200 to 300 MBq/kg (maximum: 7.4 GBq) and kidney dosimetry is used to guide dose escalation of the second cycle of LuTATE. The objective is to stay within a cumulative renal dose constraint of 23 Gy. Our approach is similar to a published LuTATE dose escalation protocol used in a phase II clinical trial in pediatric neuroblastoma currently undergoing recruitment. 1 Following the two cycles of LuTATE the patient is reassessed for further therapy.

For each cycle the patient is admitted to our radionuclide therapy room and peripheral venous access is established if the patient does not have central venous access. Ondansetron 4 to 8 mg is administered to reduce nausea prior to the amino acid infusion. An amino acid infusion (20 mL/kg of Synthamin 17 electrolyte-free solution up to 1 L) for renal protection is commenced and given over 4 hours. The LuTATE is administered IV an hour after the commencement of the amino acid infusion and given over approximately 1 hour. The patient is transferred to the nuclear medicine department after LuTATE has been administered and near the end of the amino acid infusion for dosimetry imaging. The patient is discharged after the scans are performed.

## Tumor and Kidney Dosimetry Protocol

Patient dosimetry is performed on the posttherapy LuTATE scans acquired on a GE 870 CZT gamma camera (GE Healthcare). A whole-body sweep and SPECT CT of the kidneys are performed at the end of the amino acid infusion (prior to passing urine; day 0) and then again at 24 and 48 hours post-LuTATE administration to evaluate tumoral LuTATE accumulation and to perform kidney dosimetry calculations. The scans are analyzed using proprietary GE dosimetry software (Dosimetry Toolkit) on an Xeleris workstation. 2 For each cycle of LuTATE, the day 0, 24, and 48 hours posttherapy LuTATE scans are loaded and registered to each other, then the left bronchial tumor and kidneys are segmented on both the whole-body sweep and SPECT CT, respectively, at each time point. Accurate LuTATE administered dose and timing of posttherapy scans are entered from LuTATE therapy worksheet data. The calculated tumor and kidney residence times (time-integrated activity coefficients) of LuTATE are then entered into MIRDcalc internal dose calculation software. 3 The bronchial tumor is assumed to be 100% soft tissue composition and tumor volume is estimated assuming an ellipsoid shape and using its anteroposterior, transverse, and craniocaudal dimensions measured on contrast-enhanced diagnostic CT. Using the kidneys as the sole source organs, and selecting an appropriate age and sex phantom, MIRDcalc software calculates the estimated kidney absorbed radiation dose and tumoral absorbed radiation dose in Gy. A cumulative kidney dose constraint of 23 Gy is used as an upper limit to allow escalation for further doses of LuTATE.

## Case Report

A 13-year-old female presented to her local hospital with left-sided chest pain and shortness of breath on a background of 3 weeks of fevers and weight loss. She did not have any symptoms or signs of excessive hormone production. Apart from quiescent oligoarticular juvenile idiopathic arthritis, she had no other medical conditions. A contrast-enhanced thoracic CT scan showed a 2.6 cm × 3.1 cm × 2.2 cm, rounded, contrast-enhancing mass at the left pulmonary hilum, with pulmonary collapse distal to the mass. The mass displaced multiple adjacent vessels including the left pulmonary artery, inferior lobar pulmonary artery, and left pulmonary vein, and invaded the left main bronchus. A bronchial carcinoid tumor was suspected. An (18F)FDG (FDG) PET CT was performed to characterize the malignant potential of the mass, and it showed moderately increased tracer uptake within the mass (SUVmax 6.2) in addition to FDG avid enlarged right paratracheal, left pulmonary hilar, and subcarinal lymph nodes. The increased metabolic activity raised the possibility of an atypical or high-grade lesion.

**Fig. 1:**
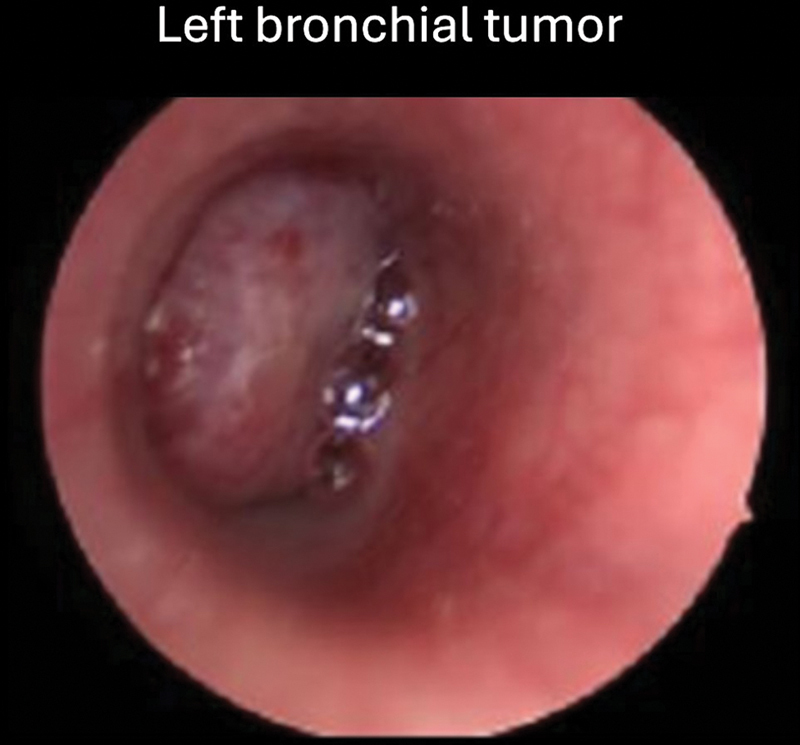
Flexible laryngobronchoscopic photo showing the endobronchial tumor component obstructing the lumen of the left main bronchus.

The patient was transferred to our institution where flexible laryngobronchoscopy demonstrated the endobronchial component of the tumor obstructing the lumen of the left main bronchus ( Fig. 1 ). Biopsy revealed a grade G1 pulmonary carcinoid with a Ki67 proliferative index of 8% raising the possibility of an atypical carcinoid tumor.

**Fig. 2:**
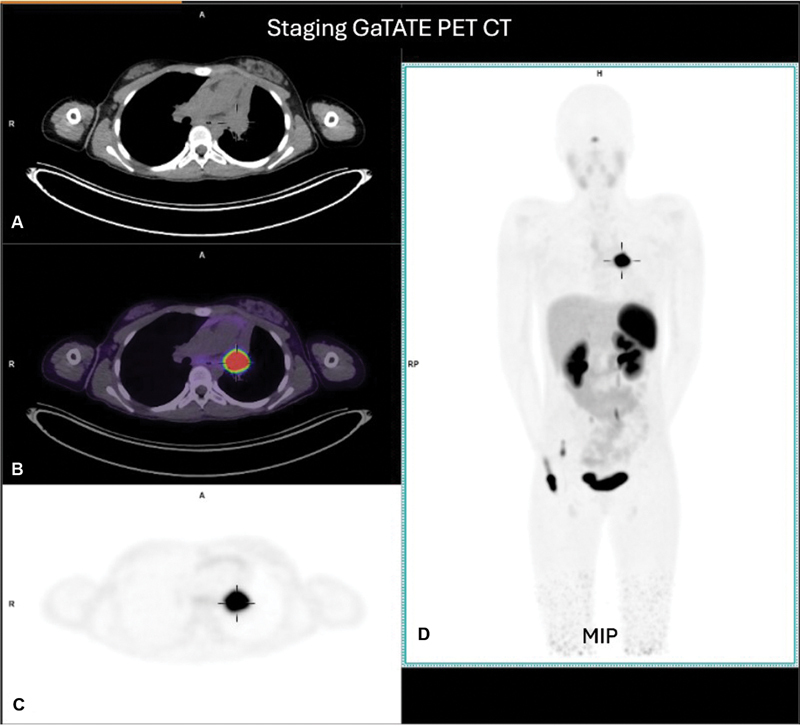
Staging GaTATE PET CT scan. Axial slices of the low-dose CT scan ( **A** ), fused PET/CT ( **B** ), and GaTATE PET ( **C** ) and whole-body MIP in the anterior projection ( **D** ) show the intensely GaTATE avid (SUVmax 40.8) left bronchial carcinoma (crosshairs). There was also low-grade uptake in the right hilar, paratracheal, and subcarinal regions concerning regional nodal metastatic disease, with physiologic tracer distribution elsewhere.

The case was discussed at a neuroendocrine tumor multidisciplinary team (MDT) meeting and a primary surgical approach including a left pneumonectomy was considered appropriate. The size and location of the tumor closely related to the left pulmonary vessels meant that definitive surgery aiming at complete resection would be associated with significant risk. Options to downstage the tumor prior to surgery were discussed, and both chemotherapy and radionuclide therapy were viable options. A GaTATE PET CT scan was performed and confirmed intense somatostatin receptor expression in the left pulmonary carcinoid tumor (SUVmax of 40.8) obstructing the left main bronchus ( Fig. 2 ). There was lower grade GaTATE activity seen in the right hilar, paratracheal and subcarinal regions concerning for regional nodal metastatic disease. The patient proceeded to neoadjuvant LuTATE therapy.

The family consulted with the nuclear medicine services to discuss the procedure, associated radiation issues, and risks related to LuTATE therapy, and consent was given to proceed. Full blood count, serum electrolytes, urea, creatinine, liver and renal function blood tests, and a 99mTc-DTPA two-sample radionuclide GFR were confirmed to be normal.

**Fig. 3:**
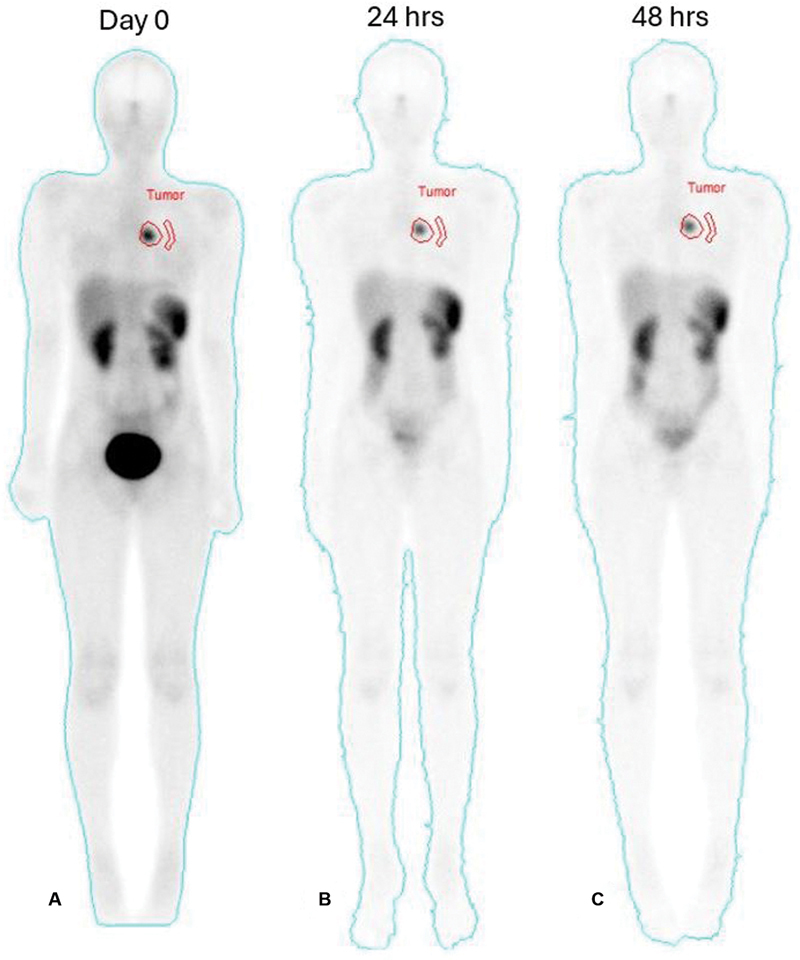
Posttherapy whole-body scans following the first cycle of LuTATE. Anterior projections of the whole-body sweeps were performed immediately following ( **A** ), at 24 hours ( **B** ) **,** and 48 hours ( **C** ) following 6.9 GBq (150 MBq/kg) LuTATE. There is intense LuTATE uptake in the left bronchial tumor and physiologic distribution elsewhere. The tumor region of interest and background region are shown in each image. Dosimetry calculations from these images estimated a 17 Gy absorbed dose delivered to the tumor.

**Fig. 4:**
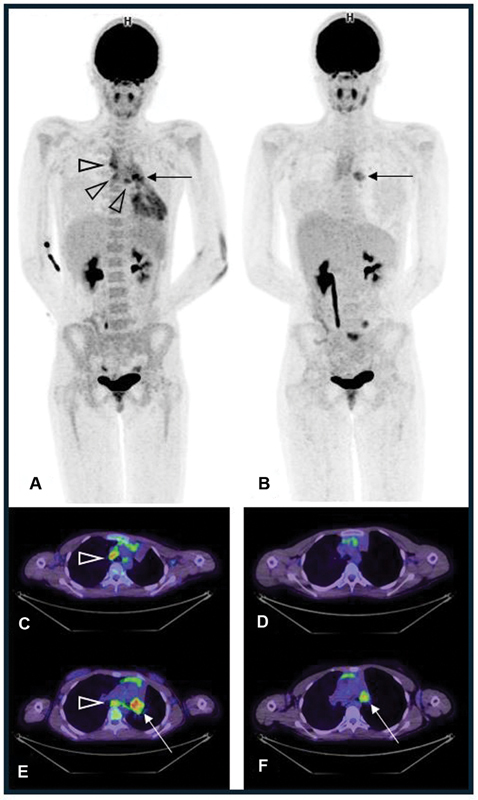
Anterior maximum intensity projection (MIP; **A** ) and axial slices of the fused FDG PET CT ( **C, E** ) before the first cycle of LuTATE; and anterior MIP ( **B** ) and axial slices of the fused PET CT ( **D, F** ) after the first cycle of LuTATE. Prior to treatment, the MIP showed moderately intense FDG uptake in the left bronchial tumor ( **A** , arrow, SUVmax 6.2) and increased uptake in the left hilar, subcarinal, and right paratracheal lymph nodes (open arrowheads). Prior to treatment, the axial fused slices show FDG avid right paratracheal lymph nodes ( **C** , open arrowhead), subcarinal uptake ( **E** , open arrowhead), and intensely FDG avid left bronchial mass ( **E** , arrow). Following the first cycle of LuTATE, the MIP shows diminished FDG uptake in the left bronchial tumor ( **B** , arrow, SUVmax 4.7) and resolution of the abnormal FDG uptake in the hilar, right paratracheal, and subcarinal lymph nodes. The axial fused PET CT slices more clearly show resolved FDG uptake in the right paratracheal region ( **D** ) and subcarinal space ( **F** ), and residual but less intense FDG uptake in the left bronchial tumor ( **F** , arrow, SUVmax 4.7).

The first empiric cycle infused 6.9 GBq LuTATE (150 MBq/kg) and the posttherapy scans showed intense LuTATE uptake in the left bronchial carcinoid tumor ( Fig. 3 ). Dosimetry calculations on the posttherapy scans estimated 17 Gy absorbed dose delivered to the tumor and approximately 6 Gy kidney exposure. The FDG PET CT scans performed after the first cycle showed diminished FDG uptake in the bronchial tumor and resolution of the hypermetabolism previously seen in the right paratracheal, left hilar, and subcarinal lymph nodes ( Fig. 4 ).

**Fig. 5:**
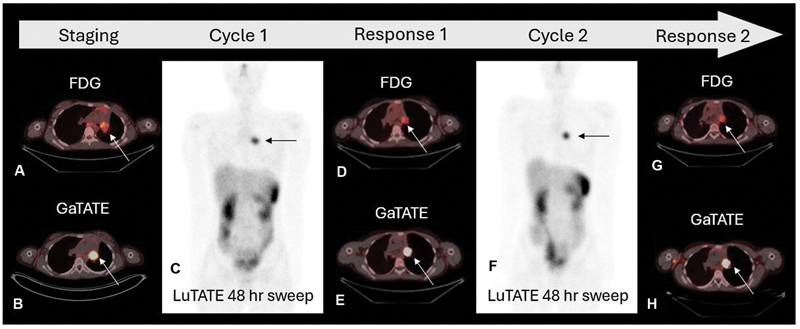
Axial fused images of the staging FDG ( **A** ) and staging GaTATE ( **B** ) PET CT, whole-body sweep following first cycle of LuTATE ( **C** ) and subsequent axial fused images of the response FDG ( **D** ) and GaTATE ( **E** ) PET CT, whole body sweep following second cycle of LuTATE ( **F** ) and axial fused images of the final response FDG ( **G** ) and GaTATE ( **H** ) PET CT. The staging PET CT scans show moderate FDG uptake ( **A** , SUVmax 6.2) and intense GaTATE uptake ( **B** , SUVmax 40.8) in the left pulmonary hilar bronchial carcinoid tumor (arrows). FDG uptake but no GaTATE uptake is also shown in the subcarinal region ( **A** ). Intense LuTATE uptake in the tumor is seen following the first cycle of 6.9 GBq LuTATE ( **C** , arrow) and dosimetry calculations estimated a delivery of 17 Gy to the tumor. The response FDG and GaTATE PET CT scans show diminished FDG uptake ( **D** , SUVmax 4.7) and persisting intense GaTATE uptake ( **E** , SUVmax 34.0) in the left pulmonary hilar tumor (arrows). Intense LuTATE uptake is again demonstrated following the second cycle of 8.2 GBq LuTATE ( **F** , arrow) where dosimetry calculations estimated delivery of a further 17 Gy to the tumor. The subsequent response PET CT scans show persisting low-grade FDG uptake ( **G** , SUVmax 4.4) and intense GaTATE uptake ( **H** , SUVmax 40.8) in the left pulmonary hilar tumor (arrows).

The progress GaTATE PET CT scan performed 3 weeks after LuTATE showed continued intense GaTATE uptake in the left bronchial mass and a second cycle of 8.2 GBq LuTATE (170 MBq/kg) was administered 4 weeks after the first cycle with dosimetry calculations estimating another 17 Gy administered to the tumor ( Fig. 5 ).

**Fig. 6:**
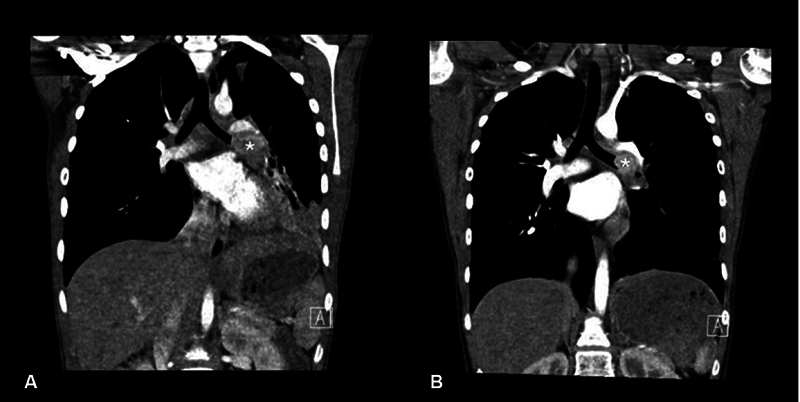
Coronal reformats of the thoracic contrast-enhanced diagnostic CT scans prior to treatment and following the second cycle of LuTATE therapy. The left bronchial carcinoid tumor is marked with an asterisk in each image. Prior to treatment ( **A** ) **,** the bronchial carcinoid tumor measures 2.6 × 3.1 × 2.2 cm (anteroposterior × transverse × craniocaudal dimensions) obstructing the left main bronchus with pulmonary collapse distal to the mass. Following the second cycle of LuTATE ( **B** ) the mass has reduced in overall size now measuring 2.8 cm × 2.5 cm × 2.4 cm (anteroposterior × transverse × craniocaudal dimensions) and the left lung has re-inflated.

The diagnostic contrast-enhanced thoracic CT scan performed 5 weeks after the second cycle of LuTATE showed evidence of a treatment response with a reduction in the size of the bronchial tumor and re-expansion of the lung distal to the tumor ( Fig. 6 ).

Bronchoscopic re-evaluation was undertaken 2 months after the second LuTATE cycle at which point a subtotal endobronchial resection was performed. LuTATE therapy conferred a treatment response based on a reduction in the size of the bronchial mass, re-aeration of the left lung, and resolution of the FDG avid hilar and subcarinal lymph nodes. Following MDT discussion local control with definitive surgery was considered necessary and further LuTATE therapy was supported in an attempt to gain further treatment response and downstage surgery to an endoscopic sleeve resection and sparing the morbidity of a pneumonectomy.

**Fig. 7:**
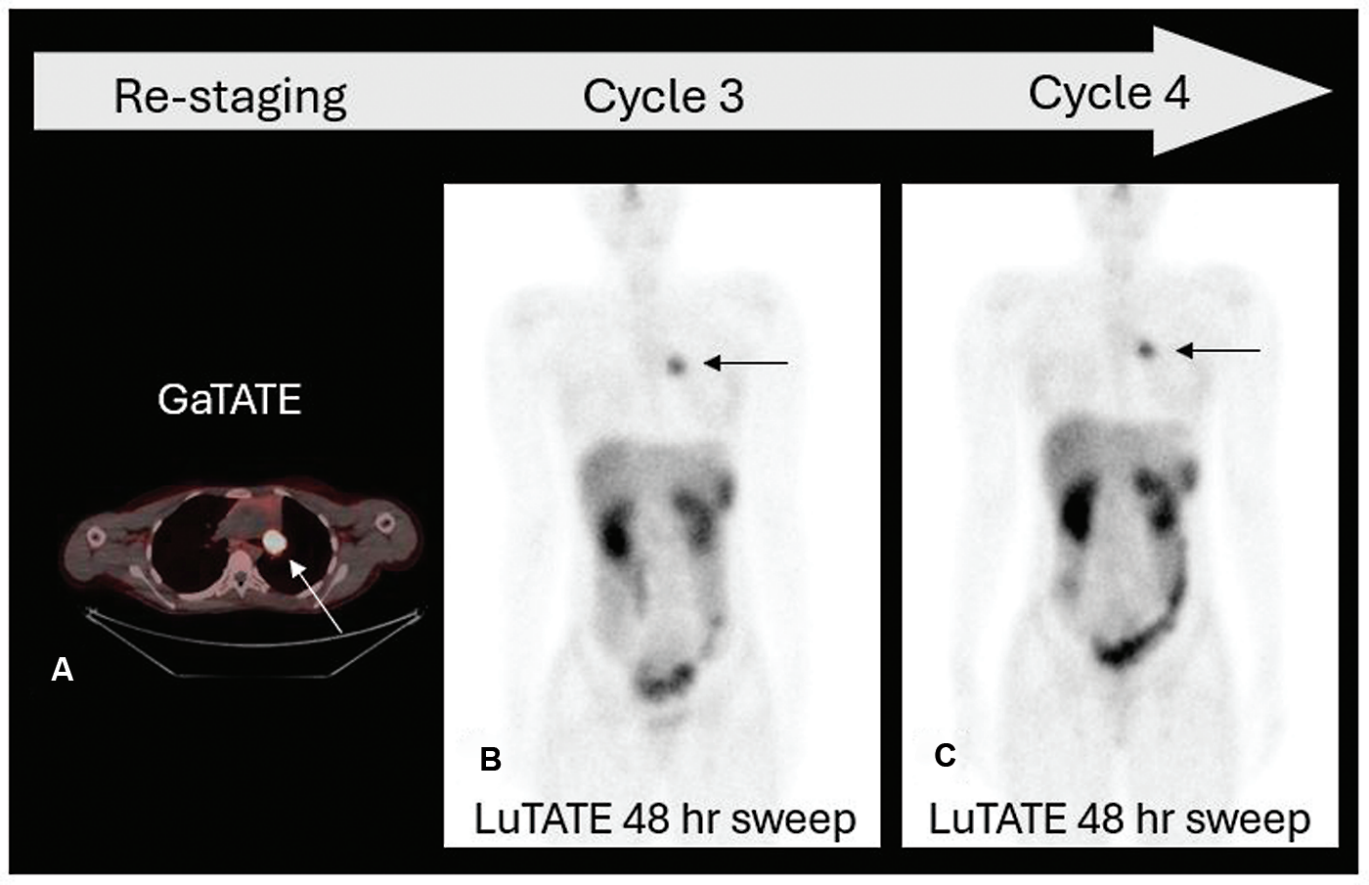
Axial GaTATE PET CT prior to further radionuclide therapy ( **A** ) and whole-body sweeps following the third ( **B** ) and fourth ( **C** ) cycles of LuTATE. Four months after the second dose of LuTATE, a GaTATE PET CT showed persisting intense GaTATE uptake in the left pulmonary hilar tumor ( **A** , arrow). Two further cycles of LuTATE therapy were administered with the intention of achieving further treatment response prior to definitive surgery. Intense LuTATE accumulation was seen following 8.3 GBq LuTATE given in cycle 3 ( **B** , arrow) and 10.0 GBq given in cycle 4 ( **C** , arrow). Tumor dosimetry calculations estimated a further 15 Gy delivered to the tumor with each cycle.

Blood tests and radionuclide GFR remained normal and repeat GaTATE PET CT confirmed intense somatostatin receptor density in the residual left bronchial mass. Four months after the second cycle of LuTATE, two further cycles of LuTATE were administered 12 weeks apart. The doses were 8.3 GBq (176 MBq/kg) and 10.0 GBq (212 MBq/kg), respectively. Dosimetry calculations indicated kidney radiation exposure to remain below 23 Gy in total, and radionuclide GFR remained normal. The post-LuTATE scans confirmed persisting intense uptake in the left bronchial tumor and no metastatic disease ( Fig. 7 ).

The fourth LuTATE cycle was planned sooner but the patient had an episode of significant emotional distress related to coping with the implication of her diagnosis and required input from psychological services to be able to receive the fourth dose of LuTATE. The fourth cycle was eventually administered 12 weeks after the third cycle. All cycles of LuTATE were administered with no complications and were well tolerated. Contrast contrast-enhanced CT scan performed 4 weeks after the final dose of LuTATE showed no significant change compared with the CT scan performed 7 months earlier following the first dose of LuTATE, and at bronchoscopy, the tumor had not shown evidence of further treatment response. The patient underwent a left pneumonectomy and histopathology showed complete resection of an atypical pulmonary carcinoid (neuroendocrine tumor grade 2) with evidence of direct extension into a peribronchial lymph node. Seven other lymph nodes were negative for involvement. Her postoperative course was uneventful, and she remains under surveillance.

## Discussion

In adults, bronchopulmonary neuroendocrine tumors make up approximately 20% of primary lung tumors of which only 2% are carcinoid tumors (10–30% of which are atypical carcinoids). 4 5 In contrast, bronchial carcinoid tumors have been reported to account for 42 to 63% of primary lung tumors in the pediatric age group. 6 7 8 9 In adult series, 5-year survival rates of up to 90% are achieved with complete surgical resection of typical lung carcinoids but atypical carcinoids have a less favorable prognosis with a 10-year overall survival of 50%. 9 10 Prognosis appears more favorable in children with overall 5-year survival rates of approximately 95%. 7 8

Pediatric bronchopulmonary carcinoid tumors are extremely rare with very few published series. 11 12 Several case reports exist in children with bronchial carcinoid tumors presenting as ectopic cushing's syndrome due to ACTH secretion from the tumor. 13 14 15 16 17 18 19 20 Abel et. al have reported the largest series of pediatric bronchial carcinoid tumors 12 and they searched a German cancer registry over a 25-year period to identify 32 cases. In this series, the vast majority presented with respiratory symptoms with two having weight loss and fever. One patient had secondary amenorrhoea due to ACTH secretion and two were asymptomatic with the tumor detected incidentally during bronchoscopy for a suspected inhaled foreign body and CT scan for follow-up of a different malignancy. Seven has atypical carcinoids. Twenty-nine patients had surgical resection, one via bronchoscope. R0 resection was achieved in 20 cases. Lymphadenectomy was performed in 25 cases with four demonstrating regional node involvement. No patients received neoadjuvant treatment and no patients received PRRT. Five-year event-free survival was 100% in typical carcinoid tumors and 83% in atypical carcinoids.

Our patient was originally thought to have a typical carcinoid based on the intense somatostain receptor expression shown on GaTATE PET CT. Up to 70% of lung typical carcinoids are expected to show high-level expression of somatostatin receptors, with decreasing frequency in atypical carcinoids. 21 However, the elevated Ki67 proliferation index raised the possibility of an atypical carcinoid and this was confirmed on histopathology following left pneumonectomy. Her prognosis remains favorable and she remains under surveillance.

PRRT with LuTATE is considered a potential therapeutic option for treating bronchial carcinoid tumors in adult guidelines. 22 Careful case-by-case multidisciplinary discussion is required to balance the relative risks and benefits of LuTATE therapy, particularly in pediatric patients where the toxicity of PRRT may be greater than in adults. In this case report the risk of inducing a second malignancy due to whole-body radiation exposure was weighed up against the potential benefit if the tumor was down-staged to allow a less morbid surgical excision. Tumor size reduction, partially relieved left main bronchus obstruction, and metabolic response of potentially metastatic regional lymph nodes are considered to have conveyed some benefit to the patient notwithstanding the patient eventually proceeded to complete pneumonectomy.

In summary, pediatric bronchial carcinoid tumors are extremely rare and may present with cushing's syndrome if the tumor is secreting ACTH. If completely resected most patients have a favorable outcome. Most are somatostatin receptor-expressing, rendering LuTATE therapy a treatment option and we have reported the first case of multi-cycle high-dose LuTATE used in the neoadjuvant setting for pediatric bronchial carcinoid. We have also demonstrated the use of dosimetry calculations to guide escalating doses of multiple LuTATE cycles while maintaining safe kidney radiation exposure. Long-term benefit by reducing the risk of recurrence is a potential but unproven benefit of using LuTATE in the neoadjuvant setting and further studies are required. It is unlikely that definitive phase III clinical trials will be possible in such a rare tumor, however, LuTATE should be considered as a treatment option in the multidisciplinary treatment planning for pediatric patients with bronchial carcinoid.
